# Worsening Lower Jaw Swelling and Pain in a Teenager: Differential Diagnosis and Management

**DOI:** 10.7759/cureus.18296

**Published:** 2021-09-26

**Authors:** Gurleen Kaur Kahlon, Kedar Tilak, Noah Kondamudi

**Affiliations:** 1 Pediatrics, The Brooklyn Hospital Center, New York, USA

**Keywords:** jaw pain, jaw neoplasm, central giant cell granuloma, brown tumor, jaw swelling

## Abstract

Giant cell granuloma is a rare, benign non-neoplastic, aggressive tumor that originates mainly from the maxilla and mandible. It affects all age groups and is more commonly seen in children. We describe a 17-year-old female that presented to the Pediatrics Emergency room with a history of right lower jaw pain. Examination revealed a bone-like buccal vestibular swelling on the lower right tooth, a bone-like lingual swelling, and a pink gingival overgrowth lesion. The biopsy of the lesion revealed a central giant cell granuloma. Tissue biopsy with histopathological examination is diagnostic and surgical excision is the gold standard of treatment.

## Introduction

Jaw pain associated with swelling is a common presentation to the ED [1]. However, it has a very broad differential diagnosis that includes trauma, infections (lymphadenitis, tonsillitis, sinusitis, mumps, syphilis, Lyme disease, jaw bone osteomyelitis), salivary gland abnormalities, dental issues (malocclusion, cysts, caries or tooth abscesses), temporomandibular joint dysfunction, osteonecrosis, autoimmune conditions (rheumatoid arthritis), bruxism (teeth grinding) and tumors (benign or malignant). We present a case of a 17-year-old patient with lower right jaw pain associated with swelling and discuss the differential diagnosis and management of this patient, who was eventually diagnosed with central giant cell granuloma.

## Case presentation

**Figure 1:**
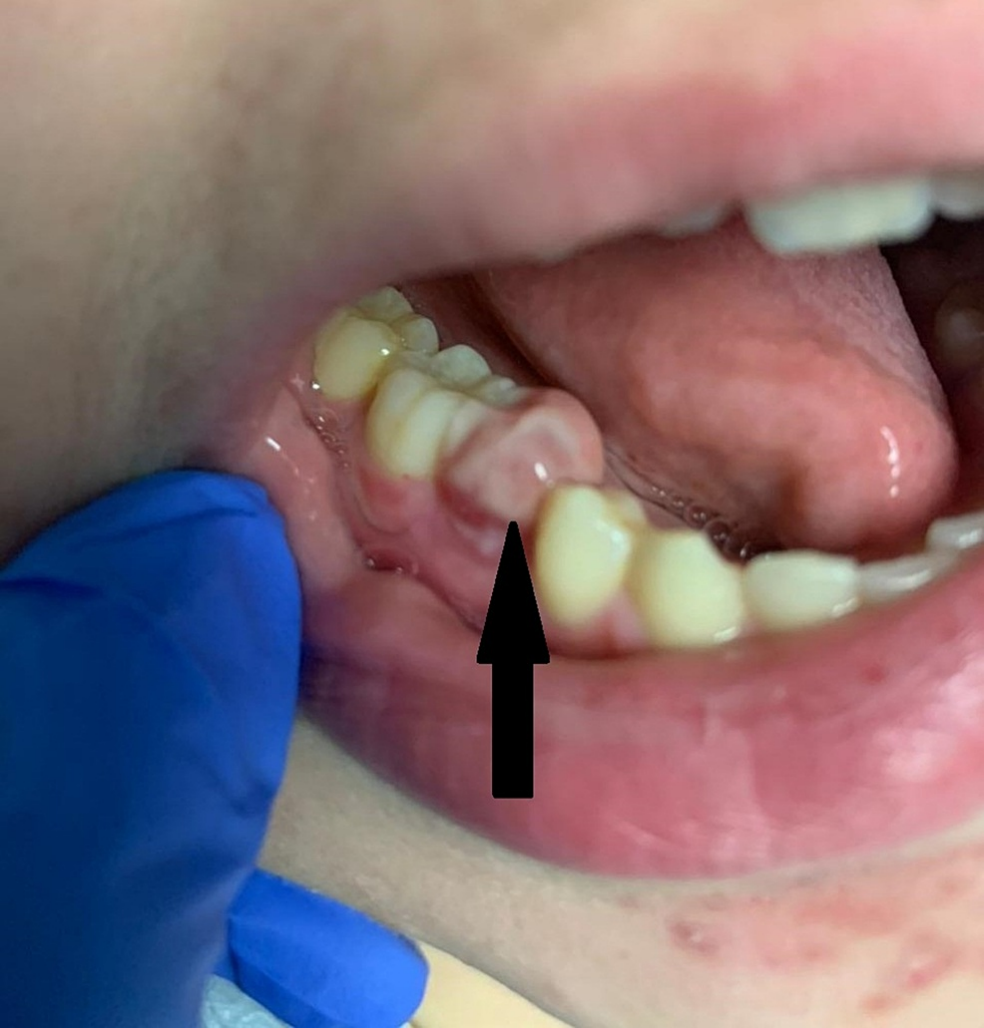
Findings on examination of oral cavity showing gingival swelling.

A 17-year-old patient presented to our pediatric emergency department with worsening lower right jaw pain for three days. The patient rated the pain to be 9/10 in intensity, dull ache in quality that worsened when brushing teeth or chewing. The pain was intermittently relieved upon taking Ibuprofen. The patient denied fever, dysphagia, headache, chest pain, chills, dysuria, oliguria, rhinorrhea, diarrhea, constipation, and abdominal pain. The patient was evaluated three weeks earlier for similar symptoms. The lower right first premolar was extracted, local biopsy performed, and the patient was discharged home on oral amoxicillin. The patient's vital signs were unremarkable: temperature 37°C (98.6°F), heart rate 70-90 beats per minute, blood pressure 119/69 mmHg, respiratory rate of 16-19 breaths per minute with oxygen saturation of 99-100% in room air. The patient's height, weight, and BMI fell between the 50th and 75th percentiles. The patient was alert, awake, and in no acute distress. There was no facial asymmetry, ecchymosis, trismus, or icterus. The conjunctiva was normal, hearing intact bilaterally with no otorrhea or neck stiffness. The rest of the systemic examination was unremarkable. Examination of the oral cavity revealed intact permanent dentition with stable and reproducible occlusion. The lower right first premolar was missing. There was a 1x1 cm tender indurated bone-like buccal vestibular swelling on the lower right mandible adjacent to the missing tooth site. A 5mm x 5mm non-fluctuant pink gingival overgrowth lesion was visible at the lower right first premolar site. There were no mobile teeth, mobile maxillary or mandibular bony segments, step deviations, tongue deviation or protrusion, or intra-oral lacerations (Figure 1).

The patient was alert, awake, and in no acute distress. There was no facial asymmetry, ecchymosis, trismus, or icterus. The hearing was normal bilaterally as assessed by Webber’s and Rinne tests, no otorrhea or neck stiffness and the conjunctiva was normal. The systemic examination was unremarkable. The initial lab data is shown in Table 1.

**Table 1:** Laboratory data for the patient.

Test		Results
CBC	TWBC	8800
	Neutrophils	56%
	Lymphocytes	33%
	Monocytes	9%
	Hemoglobin	14.5 mg/dL
	Platelets	243,000/cmm
Coagulation profile	aPTT	24.4 sec
	PT	10.9 sec
	INR	1.0
Electrolytes	Sodium	141 mmol/L
	Potassium	4.2 mmol/L
	Chloride	105 mmol/L
	CO_2_	27 mmol/L
	Creatinine	0.8 mg/dL
	BUN	10 mg/dL
	Glucose	90 mg/dL
	Calcium	9.5 mg/dL
COVID PCR and antibody tests		negative

**Figure 2:**
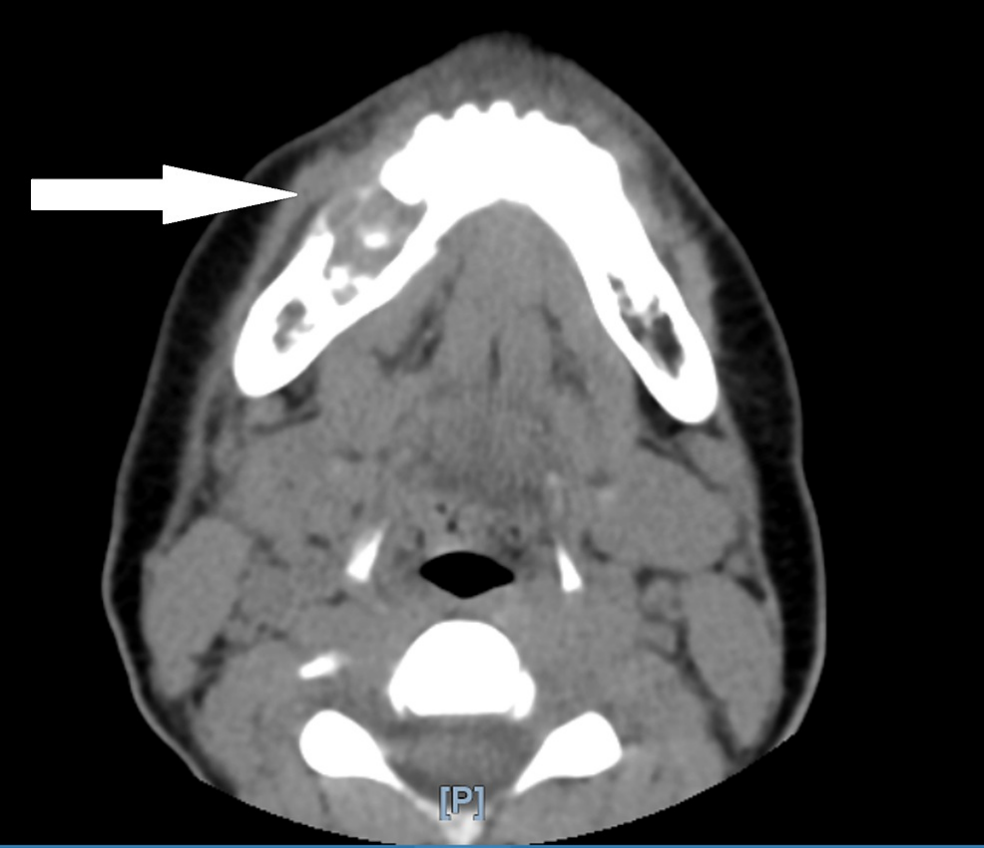
CT scan of the maxillofacial area without contrast showing lytic lesion of the right mandible.

**Figure 3:**
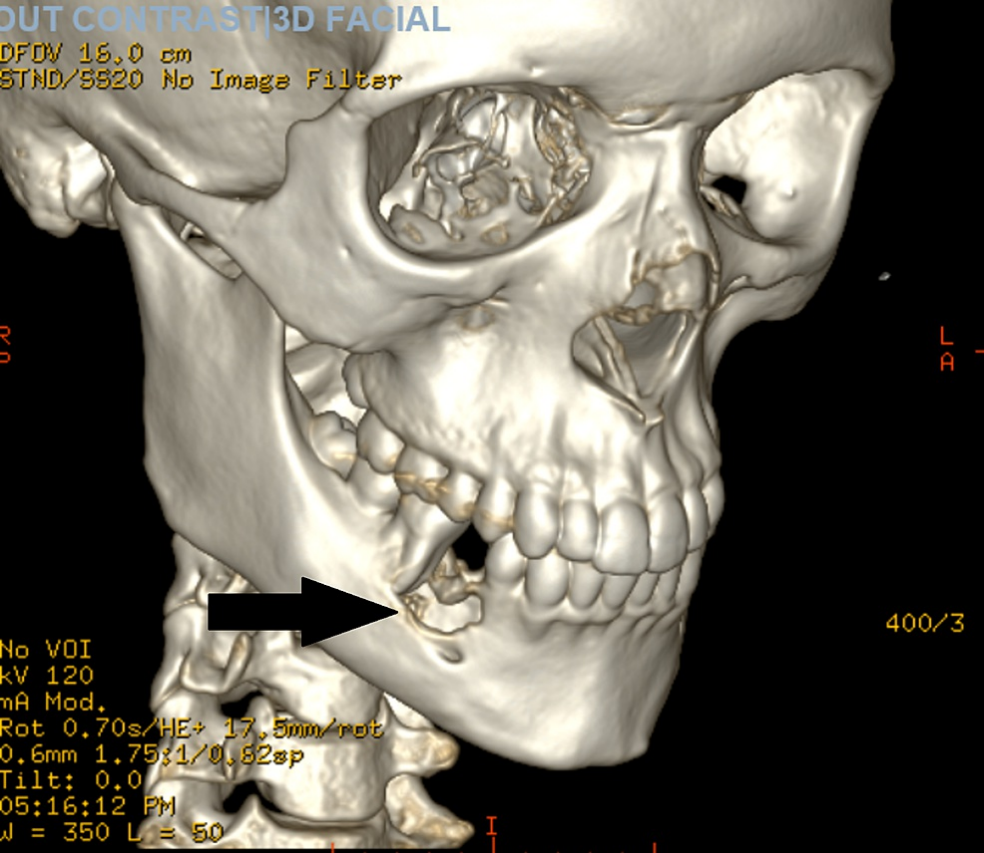
CT scan of the maxillofacial area without contrast with 3D recon showing lytic lesion of the right mandible.

The oral surgery team was consulted and a CT scan of the maxillofacial area without contrast with 3D reconstruction was recommended. CT scan showed an expansile lytic lesion centered in the body of the right mandible with erosion through both the inner and outer cortex of the mandible measuring approximately 2.2 x 1.7 x 1.7 cm. The erosion was centered on the roots of the right lower premolar teeth and first molar tooth with the destruction of the second premolar tooth. These findings were consistent with the diagnosis of giant cell granuloma (Figure 2 and 3).

**Figure 4:**
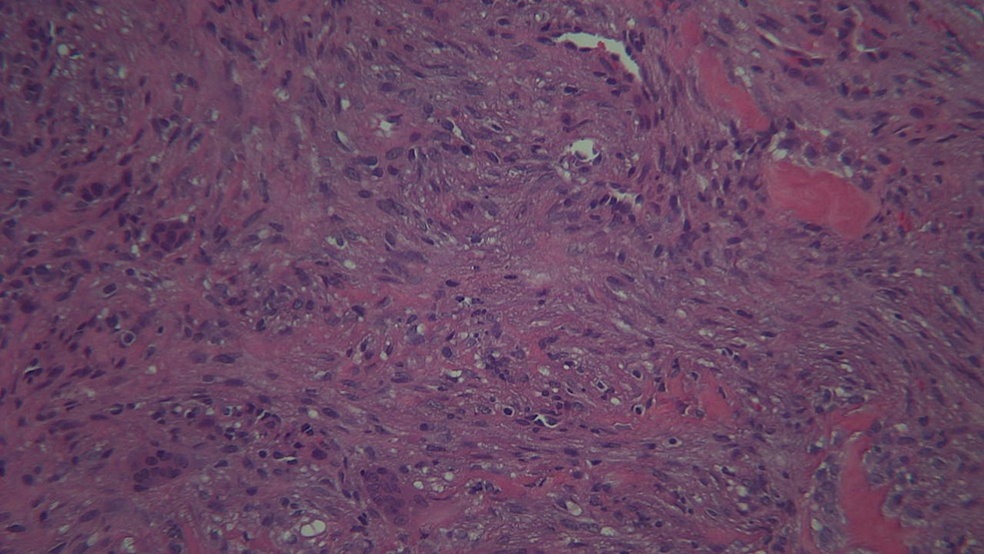
Histology slide showing clusters of foreign body-type giant cells in a hemorrhagic and well-vascularized, fibroblastic, loosely arranged stroma with lymphocytes, plasma cells, histiocytes, and osteoid trabeculae distributed throughout.

The biopsy report showed clusters of foreign body-type giant cells in a hemorrhagic and well-vascularized, fibroblastic, loosely arranged stroma with lymphocytes, plasma cells, and histiocytes distributed throughout. Osteoid trabeculae were also noted (Figure 4).

The histology report also stated that the lesion was a CGCG, indistinguishable from the giant cell lesion of hyperthyroidism (Brown Tumor). Normal serum levels of parathyroid hormone (61.9 pg/mL), calcium (9.7 mg/dL), phosphorus (4.5mg/dL), and alkaline phosphatase (83 U/L) ruled out the diagnosis of giant cell lesion of hyperthyroidism. The oral maxillofacial surgical intervention consisted of extraction of lower second premolar tooth, enucleation, and curettage of the right posterior mandibular lesion with debridement of the mandible followed by bone graft with particulate bone and platelet-rich fibrin. The patient was started on clindamycin 40mg/kg/day every six hours and made an uneventful recovery. The surgical specimen's pathology revealed a benign fibro-endothelial cell population with inflammatory giant cells and hemosiderin/extravasated red blood cells. The bacterial, fungal, and acid-fast bacilli (mycobacterium) cultures showed no growth. The patient was discharged home with close outpatient follow-up.

## Discussion

Jaffe was the first to describe giant cell reparative granuloma (GCRG) in 1953 as a rare, benign, non-neoplastic aggressive tumor, almost exclusively seen in the maxilla and the mandible [2]. The World Health Organization (WHO) defines CGCG as an intraosseous lesion made of cellular fibrous tissue, which contains multiple foci of hemorrhage, aggregations of multinucleated giant cells, and some trabeculae of woven bone [3]. The incidence of CGCG is around 0.0001%, and it accounts for 7% of all benign tumors of the mandible [4]. They present with a higher frequency in the mandible than maxilla, with a relative proportion ranging from 2:1 to 11:9 [5]. It usually affects younger patients below age 30 years with a peak age incidence range between 10 to 25 years and an apparent predilection for females [6]. Waldron and Shafer reported that 74% of patients were below 30 years of age among their 38 cases, with only 16% younger than ten years old [7].

The etiology of CGCG is unknown. The suggested theories of tumor origin include trauma, inflammatory foci, vascular mishap, or genetic predisposition [8,9]. The most common cause is post-traumatic intraosseous hemorrhage, but these are also known to occur spontaneously without any history of trauma [9].

CGCG has two main subtypes- aggressive and non-aggressive, based on their clinical and radiographic characteristics. The non-aggressive CGCG is the most common one and presents as a painless, slow-growing lesion that expands into the cortical bone. The aggressive central giant cell granulomas occur mainly in younger patients, rapidly grow in size (greater than 5 cm) with root resorption and tooth displacement. This leads to malocclusion, cortical bone thinning, or perforation. Aggressive lesions are also associated with recurrence even after surgical curettage [10].

Clinically, CGCG can be asymptomatic (if sluggish, indolent, slow growth) or present with worsening pain and swelling (if aggressive) causing rapid hollowing out of bone with cortical expansion, thinning, and perforation, root resorption and displacement of adjacent structures including teeth and nerves [8].

Radiographically, CGCG is a solitary lesion presenting as a multilocular radiolucency with scalloped margins and a honeycomb or soap bubble-like appearance [8]. This appearance can be similar to those seen in other bone tumors such as giant cell tumors, brown tumors of hyperparathyroidism, aneurysmal bone cyst, ameloblastoma, ossifying fibroma, odontogenic myxoma, and sarcomas [11]. In addition, giant cell granulomas associated with genetic disorders such as cherubism, Noonan syndrome, neurofibromatosis, and arteriovenous malformations can present with a similar soap bubble or honeycomb appearance with scalloped margins [10].

Giant cell tumor (GCT), which is histologically and immunohistochemically indistinguishable from CGCG, mainly affects the long bones like the femur and tibia (accounting for more than 50% of GCTs), and spine (12%-30%) [12]. In contrast to the CGCG, the GCT is considered truly neoplastic [13]. Malignant transformation in CGCGs is a rare phenomenon [14], but Bertoni et al. reported malignancy of the bone in 1.8% of the cases with GCT. These can be primary or secondary, and can include giant cell-rich osteosarcomas, fibrosarcomas, and malignant fibrous histiocytomas [15].

It is hard to differentiate brown tumors of hyperparathyroidism from CGCG histologically. However, the determination of serum calcium, phosphate, and parathormone assay can point to the proper diagnosis. Brown tumors can occur in 4.5% of patients with primary hyperparathyroidism and 1.5-1.7% of those with secondary hyperparathyroidism [16]. Most of these patients are asymptomatic, and hypercalcemia, hypophosphatemia, and increased alkaline phosphatase levels in the blood are often discovered incidentally during routine laboratory testing [17]. In addition to this, any skeletal bones may be affected with brown tumors, including the craniofacial bones, and these tumors can be the first clinical sign of hyperparathyroidism [16,17].

Different modalities have been tried in the past to manage CGCG depending on the type, size, and location. These include surgery, cryotherapy, radiation, antiangiogenic therapy using subcutaneous alpha-interferon, intralesional steroids (triamcinolone acetonide), or calcitonin and tyrosine kinase inhibitors (Imatinib) [2,9,10,12].

Surgery is currently the mainstay treatment of CGCG, especially with the aggressive variant. The most preferred surgical methods include enucleation, curettage with or without chemical cauterization, peripheral ostectomy, segmental resection, or en bloc resection. Nevertheless, the gold standard is excision by curettage with the removal of peripheral bone margins [2,8,18,19]. Enucleation and curettage can preserve the cortex of the mandible and inferior alveolar nerve but has been associated with a recurrence rate of 30-70% in aggressive CGCG variants. Segmental resection has the lowest recurrence rate [20]. When used in combination with cryotherapy, curettage can also decrease the rate of recurrence, especially in nonfacial bone [12].

Intra-lesional steroids inhibit bone resorption by osteoclasts in marrow cultures and may have several advantages over surgery. These include ease of administration, minimal invasiveness, less disfigurement or damage, short duration of treatment and hospital course, minimal side effects, and the option to treat surgically at an optimal time. Conversely, disadvantages of steroid therapy include long treatment time, poor patient compliance, and steroid-associated systemic side effects. In addition, in young children, surgical treatment can cause damage to primary teeth or the developing permanent tooth or gingiva and can be disfiguring compared to the non-surgical method [9].

Early diagnosis, prompt intervention, and complete removal of CGCG lesions result in an excellent prognosis and low recurrence rates [8].

## Conclusions

Jaw pain with mandibular swelling has a broad differential diagnosis and requires careful clinical assessment and appropriate workup. CGCG is a locally invasive benign tumor diagnosed with tissue biopsy after ruling out brown tumor of hyperparathyroidism. Early surgical intervention with excision, enucleation, and curettage results in an excellent prognosis.
